# New valproate regulations, informed choice and seizure risk

**DOI:** 10.1007/s00415-024-12436-8

**Published:** 2024-06-19

**Authors:** Heather Angus-Leppan, Rachel Arkell, Lance Watkins, Dominic Heaney, Paul Cooper, Rohit Shankar

**Affiliations:** 1 Present Address: University of East London, Stratford, E15 4LZ UK; 2 Kent Law School, University of Kent, Canterbury, CT2 7NS UK; 3 University of South Wales, Pontypridd, UK; 4 National Hospital for Neurology and Neurosurgery, UCLH NHS Foundation Trust, Queen Square, London, WC1N 3BG UK; 5 University of Manchester, Manchester, UK; 6 Present Address: Peninsula School of Medicine, University of Plymouth, Drake Circus, Plymouth, PL4 8AA UK; 7 Royal Free London, Pond Street, London, NW3 2QG UK; 8 Centre for Reproductive Research and Communication, British Pregnancy Advisory Service (BPAS), London, UK; 9 University of Plymouth, Plymouth, UK; 10 Manchester Centre for Clinical Neurosciences, Greater Manchester, Salford Royal Hospital, Stott Lane, Salford, M6 8HD UK

**Keywords:** Valproate, Medicines and Healthcare products Regulatory Agency, Antiseizure medications, Antiepileptic drugs, Congenital malformations, Neurodevelopmental abnormalities

## Abstract

Valproate is the most effective medication for generalised epilepsies, and several specific epilepsy syndromes. For some people, it will be the only medication to establish seizure remission, and withdrawing it carries risks of seizure recurrence and Sudden Unexpected Death in Epilepsy (SUDEP). It is also of proven efficacy for bipolar disorder and migraine prevention. Guidelines based on observational and epidemiological studies stress that maternal valproate related teratogenicity and neurodevelopmental effects are significantly higher than for other antiseizure medications (ASMs). It should, therefore, only be used if other medications are ineffective and after balancing the teratogenicity risk. Regulatory restrictions have changed prescribing practices and reduced valproate use. The number of other medications that must be trialled in the different conditions for which valproate has effectiveness and the consequences of the lack of efficacy of those drugs leading to significant harm including death remains unexplored. Risk minimisation measures (RMMs) for valproate, chiefly Pregnancy Prevention practices (PPP), consider foetal risk and not risk to people living with epilepsy. In the United Kingdom (UK), limitations relating to valproate use in all people < 55 years commenced in January 2024. While the evidence in child-bearing women is not disputed, the data in males are based on animal models, case reports, and one commissioned, unpublished, non-peer reviewed report unavailable to the UK public, stakeholder charities or professionals. Evidence suggests that 30–40% of people switching from valproate have breakthrough seizures. Thus, an estimated 21,000–28000 people in the UK will imminently be exposed to the potential hazards of breakthrough seizures, including death. There is little government investment in monitoring the effects of these changes to valproate prescribing on patient health and quality of life. This review summarises the history of valproate regulation, evidence underpinning it and argues how the latest regulations in the UK do not align with the country’s medical regulatory bodies ethical principles nor with the *Montgomery* principles of informed patient choice and autonomy. It dissects how such regulations infringe Common Law principles, nor give due regard for patient outcomes beyond reproduction. The paper looks to provide recommendations to redress these concerns while appreciating the core need for such governance to emerge in the first place.

**Supplementary Information:**

The online version contains supplementary material available at 10.1007/s00415-024-12436-8.

## Introduction

This review summarises the history of valproate (sodium valproate) regulation related to adult epilepsy, indications, risks and benefits of valproate for epilepsy. Evidence is graded A–E [1]. The ethical and legal problems with the current Medicines and Healthcare Products Regulatory Agency (MHRA) regulations are discussed, along with circumstances where patients or their representatives may choose valproate. Recommendations are made to re-align focus on the care of people wanting to take valproate after informed consent, and the monitoring of those patients avoiding it.

## History of regulation

Advocacy groups maintain that the government, pharmaceutical industry, and clinical responses have been inadequate and too slow, to valproate (VPA) teratogenicity reported in animals since the 1970s and in humans since the 1980s [2]. Since the 1990s, the consensus hardened that the teratogenic effects of VPA were greater than for other older and newer alternatives ASMs (including lamotrigine and later levetiracetam) [3]. Since 2014, valproate has not been recommended for use in the UK for girls and women of childbearing potential unless other treatments are ineffective or not tolerated. It is no longer listed as the first-choice treatment for women with genetic generalised epilepsies, despite its superior efficacy to all other ASMs [4]. Risk minimisation measures (RMMs) to reduce teratogenicity and/or developmental disorders mandated the Pregnancy Prevention Programme (PPP): that is “user independent” contraception (intrauterine devices or subdermal progesterone only implant) for women taking valproate [5]. This created a gender divide in prescribing practice based on guidelines and increasing statutory restrictions [6]. In England, the number of pregnant women prescribed valproate in a 6-month period fell from 68 women in April to September 2018, to 17 women in October 2021 to March 2022 [7]. The MHRA projects a 11% risk of birth defects and a 30–40% risk of neuro-developmental disabilities [7], but there are no published data on the outcomes of these pregnancies, where dose-adjustment and other mitigations may have been effective. In a recent study, approximately 11% of women with epilepsy self-reported being on valproate [8]. Concerns exist about the blanket nature of including all women of childbearing age with epilepsy [6, 9–12], and for those who lack the ability to have an informed consensual relationship especially those with intellectual disability [9, 10, 12]. It was recognised that the situation is complex especially regarding people with intellectual disability and UK practices were out of kilter with other parts [9–13].

In November 2023, the MHRA announced new RMMs bringing restrictions to all people under the age of 55 years [14]. As shown in Box 1, the emergence of the mechanisms and operational programme for these new measures are unclear. Some of the critical source data for this extension have not been shared with health professionals despite multiple requests to the MHRA, including two Freedom of Information requests from amalgamated epilepsy charities [15]. The MHRA appears to have implemented changes based on a report that was not suitably peer-reviewed and published. The MHRA recorded that “errors have been identified in the study that may impact on the results” requiring further analysis [15]. This leaves professionals and charities in the UK with the impossible task of supporting people with epilepsy in informed decision-making without the information [13, 15].

Box 1: Evolution of regulatory authority statements about valproate use **New Zealand Medicines and Medical Devices Safety Authority** 2014 [16].

 Valproate is contraindicated in pregnancy.

 Valproate should not be used in women with childbearing potential unless other treatments are ineffective or not tolerated.

**Joint Task Force of International League Against Epilepsy, Commission on European Affairs, and European Academy of Neurology **(adopted China and elsewhere) 2016 [17].

 Where possible valproate should be avoided in women of childbearing potential.

 Need to balance teratogenic risks of valproate and treatment alternatives, the importance of seizure control, and risks to patient and fetus from seizures, and the effectiveness of valproate and treatment alternatives for the different epilepsies.

 Shared decision between clinician and patient and, where appropriate, the patient’s representatives.

**US Food and Drug Administration** 2016 [18].

 *Epilepsy and bipolar disorder*: category D—potential benefit for pregnant women may be acceptable despite the potential risks, should be given to pregnant women and those of childbearing potential only if other medications have not controlled the symptoms or are otherwise unacceptable.

 *Migraine*: category X, indicating the risk to pregnant women clearly outweighs any possible benefit.

**French National Agency for the Safety of Medicines and Health Products** 2017 [19].

 *Bipolar disorder*: Valproate banned for women and girls of childbearing age without effective contraception.

 *Epilepsy*—avoid in girls, adolescents, women of childbearing age, and pregnant women, except in cases of treatment failure.

**UK Medicines and Healthcare Products Regulatory Agency** 2018 [20].

 Valproate should only be used in women and girls of childbearing potential if nothing else works.

 This group must follow the pregnancy prevention programme.

**European Medicines Agency’s Pharmacovigilance Risk Assessment Committee** 2018 [21].

 *Migraine or bipolar disorder*: Valproate must not be used in pregnancy.

In female patients from the time, they become able to have children—valproate must not be used unless pregnancy prevention programme conditions are met.

 *For epilepsy*: Valproate must not be used in pregnancy. However, it is recognised that for some women with epilepsy it may not be possible to stop valproate and they may have to continue treatment (with appropriate specialist care) in pregnancy.

In female patients from the time they become able to have children—valproate must not be used unless the conditions of the new pregnancy prevention programme are met. This involves pregnancy tests before starting and during treatment as needed, counselling patients about the risks of valproate treatment, effective contraception throughout treatment, reviews of treatment by a specialist at least annually, and completing a new risk acknowledgement form at each review.

**UK Medicines and Healthcare Products Regulatory Agency** December 2022 [22].

 The Commission on Human Medicines (CHM) has advised that no one under the age of 55 should be initiated on valproate unless two specialists independently consider and document that there is no other effective or tolerated treatment.

**UK Medicines and Healthcare Products Regulatory Agency** August 2023 [23].

 “The CHM has advised that further guidance in respect of risks in children of men taking valproate should be based upon data that are accurate and complete. Any further guidance will be communicated to patients and healthcare professionals as soon as possible.” (unpublished Study V, remains unpublished).

**The European Medicines Agency (EMA) **August 2023 [24].

 “Currently evaluating the results of a study which may indicate a risk of neurodevelopmental disorders of 5.6–6.3% for children born to fathers treated with valproate, compared with 2.5–3.6% for children born to fathers treated with lamotrigine or levetiracetam. The results may change following multiple further analyses the EMA has now requested from the pharmaceutical companies involved.” (unavailable, unpublished Study V).

**New Zealand Medsafe** December 2023 [25].

 Use of sodium valproate in people who can father children within the 3 months prior to conception may increase the risk of neurodevelopmental disorders in the child.

 The potential risks to children fathered more than 3 months (the time taken for new sperm to be formed) after stopping sodium valproate are unknown.

 If stopping sodium valproate, continue effective contraception for 3 months.

There may be corrections to the retrospective observational study results following reanalysis of one data set (unavailable, unpublished Study V).

**Medicines and Healthcare Products Regulatory Agency **2024, 31 January 2024 [26].

 Valproate must not be started in new patients (male or female) < 55 years, unless two specialists independently document that there is no other effective or tolerated treatment; or compelling reasons that the reproductive risks do not apply.

## VPA indications

VPA is licensed to treat all forms of epilepsy [27]. It also has FDA indications for bipolar disorder (including acute mania and mixed episodes) and migraine prevention [28]. Outside licence, it is used occasionally to treat various pain syndromes, although evidence of its benefit in these contexts is lacking [29]. Potential adjuvant therapy roles in conditions such as HIV and cancer, and in neuroprotection (stroke and neurodegenerative diseases), possibly through histone deacetylase inhibition, are under exploration [27].

It became clear from post-licencing clinical experience and comparative randomised-controlled studies published during the 1980s and 1990s that VPA offered clinically significant advantages in seizure control compared with other medications for patients with generalised tonic–clonic seizures (GTCS), which are the hallmark of many of the genetic (idiopathic) generalised epilepsies [30]. A randomised multicentre trial showed that valproate was the most effective medication for generalised epilepsies [31]. Until recently, range of academic and professional guidelines, including NICE, recommended valproate as “first-choice” treatment for this group [32]. Valproate is also considered effective for a number of paediatric epilepsy syndromes, such as Dravet’s syndrome [33].

## Sexual health, reproduction and valproate

Studying sexual health is challenging given the lack of standardisation of definitions and outcome measures, and the wide variability that exists across age, cultures and socioeconomic settings [34]. Chronic conditions, including epilepsy, can affect sexual function and fertility in many ways. For people with epilepsy, many inter-related factors can contribute to sexual dysfunction [35]. Those with comorbidities (particularly Intellectual Disability (ID) and psychiatric problems) more severe epilepsy, certain seizure types, and those who are socially deprived are more likely to have difficulties [34]. Fertility levels are reported as lower for people with epilepsy in small, detailed surveys [36] and large population studies [37]. Nevertheless, evidence suggests no direct relationship between sex hormone levels and sexual happiness, with high levels of sexual satisfaction occurring in some people with abnormal hormonal profiles [34, 35]. Men in relationships who have epilepsy report the same level of sexual satisfaction as those in relationships without epilepsy [34, 35].

Valproate may indirectly increase sexual happiness through seizure freedom, which is the main determinant of quality of life in epilepsy and through mood stabilising effects [34]. Indirect negative effects of valproate on sexual function include weight gain [38], insulin resistance with associated increased risk of hypogonadism in males [38], and polycystic ovarian syndrome in women^.^ [38, 39].

## Women

Female sexual dysfunction increases with age and worldwide is reported as 41% [40]. ASMs, including VPA, epilepsy, chronic illness all have reported associations with sexual dysfunction, as do socioeconomic deprivation (which correlates with severity of epilepsy), stress and psychiatric illness [34, 41]. The focus of sexual health research in epilepsy has been on reproduction.

There are no current, or planned, randomised controlled trials of ASM use in pregnancy. Observational data derived from population studies, pregnancy registers, and case–control studies in people with epilepsy are the principal evidence for all guidance on this subject. Cochrane reviews conclude that methodological flaws do not affect the assertions of the risks of valproate [42]. A pooled analysis reported major congenital malformations (defined as an abnormality of an essential anatomical structure that substantially interferes with function or requires major intervention) [3] in 6.1% (range 4–10%) [43] of children whose mothers took ASMs during pregnancy, compared with 2.8% in children of untreated women with epilepsy and 2.2% in the general population [44]. Registries [44–48] report rates of major malformations for valproate ranging from 6.7% in the UK and Ireland Epilepsy and pregnancy registers which includes a third of relevant pregnancies [45], 10–13.8% in the Australian Pregnancy Registry, which includes 1 in 12 relevant pregnancies [47]. There are few prospective reports for indications other than epilepsy. The Australian registry captured nine children of women who were taking valproate for indications other than epilepsy, one of whom had a malformation (cleft palate) [49]. Some major malformations (hypospadias, cleft palate, polydactyly, cardiac defects) are treatable, but many leave children dependent, with serious lifelong disability.

Maternal valproate is also associated with neurodevelopmental disorders. These are more difficult to quantify on a shifting backdrop of increasing diagnosis, expanding definitions and potential ascertainment bias [50]. In 2016, 1.85% of children in USA were estimated to have autism compared to 0.67% in 2000 [51]. Scandinavian population studies of maternal valproate exposure report an increased diagnosis of autism (2.5% versus 0.5% in the general population [52]) and attention–deficit/hyperactivity disorder [53]. The mean IQ of children at 6 years with maternal valproate exposure (49/62 children from 25 centres in the UK and US, countries with different educational systems), fell within the normal range at 97 (95% confidence interval 94–101), but lower than for those with maternal lamotrigine exposure (108, CI 105–110). Mean maternal IQ of mothers taking valproate was 96 (CI 92–100), lower than the mean maternal IQ of mothers taking lamotrigine (101, CI 98–104) but all within the normal range [53]. Guidelines and MHRA regulatory statements quote neurodevelopmental problems in 30–40% of offspring of mothers taking valproate [7, 14, 22, 23]. These figures are higher than estimates from population studies of neurodevelopmental disorders in 6.5% of children of mothers taking valproate compared to 2.4% in mothers with untreated or resolved epilepsy [54, 55].

## Men

There is little data on the impact of epilepsy and its treatment with ASMs on sexual function in men, with no Grade A evidence or registry studies [1, 34]. Sexual dysfunction is variably defined, and surveys in the general male population estimate the range is from 9.6% of 594 in Norway [57] to 31% of 1410 in US [58] and is reported in two-thirds of men with refractory epilepsy [57]. Epilepsy and ASMs have direct and indirect influences on sexual function and dysfunction including hyposexuality, erectile dysfunction, hypersexuality or impaired fertility (Table 1).

**Table 1:** Sexual function in men with epilepsy and the role of VPA containing medicines (adapted from Watkins and Angus-Leppan, 2020 [34])

Factors influencing sexual function	Evidence for role of Valproate containing medicines
Epilepsy aetiology	There may be a direct genetic influence on sexual function (e.g., MTHFR Polymorphisms) Valproate (and other ASMs) can interfere with folic acid metabolism The evidence base for teratogenic effects is in women taking valproate, there is little published evidence of paternal teratogenic risk (see text)
Seizures	Seizures from an early age, high seizure burden, and treatment resistance may impact on sexual function Valproate has best efficacy in the treatment of generalised genetic epilepsies Seizure freedom is the main determinant for quality of life in people with epilepsy
Co-morbid medical conditions	Valproate can be associated with weight gain and insulin resistance The metabolic syndrome is associated with lowered sexual function across a range of domains
Co-morbid psychiatric conditions	Valproate has a positive mood profile, and is used to treat depressive and manic symptoms, reducing the need for polypharmacy, potentially improving sexual function
Anti-seizure medication (ASM)	The treatment of seizures improves quality of life and improves sexual function ASMs in general (including VPA) may affect sex hormone functioning, the sex hormone axis, and sperm abnormalities

ASMs potentially reduce male fertility through hormonal effects, erectile dysfunction or reduced sperm quality. Teratogenicity or neurodevelopmental problems in offspring of men could be possible through direct transmission of ASMs to the embryo, or through genetic or epigenetic mechanisms. The latter has the potential to cause transgenerational damage. These are discussed, along with evidence from population studies.

## Valproate and male fertility-hormonal effects

Data on reproductive hormones in men taking valproate are contradictory and limited to small numbers (Grade C evidence) [34, 59–61, 66, 69]. A review totalling 444 patients and 398 controls found that valproate had fewer hormonal effects than enzyme-inducing ASMs [59]. Another review found an association between valproate use in men and lowered testosterone, *p* = 0.04 [60]. Contradictory findings were found in a controlled study in 90 men for whom in those treated with valproate, 57% had elevated serum androgens (*n* = 12, *p* 0.001) as compared with 20% in those taking carbamazepine and 8% in controls [61]. In another study, of 70 males with epilepsy treated with VPA (age-matched to 70 controls) (age range 7–20 years) androgen levels were elevated in 45 (64%, *p* = 0.0006) particularly in prepubertal patients (*p* = 0.0003) [62].

### Erectile dysfunction

Valproate reduced rodent erectile dysfunction and penile fibrosis in a small study [63] and is trialled as a neuroprotective agent to reduce the risk of erectile dysfunction requiring radical prostatectomy [64]. Population surveys report erectile dysfunction in 2% of healthy men < 40 years and up to 80% of men > 80 years [65]. 42.5% of 80 men with epilepsy reported erectile dysfunction using the self-administered scale [66] with similar rates (38.6%) in an uncontrolled hospital cross-sectional survey in men with neurologic disability [67] (Grade C evidence). Self-reported reduction in erectile function was noted by men taking valproate (*n* = 32) and levetiracetam (*n* = 30) compared to controls without epilepsy on no medication, along with reduced sperm motility in both groups; and increased abnormal sperm in the valproate group [68].

### Valproate and male fertility—sperm

Pharmacological modelling estimates that concentrations of valproate are < 25,000 times lower for male seminal fluid than the equivalent dose of a single oral 500 mg tablet for women. Therefore, paternal valproate ingestion does not cause adverse effects to the embryo via direct passage [69]. The applicability of animal studies to human experience is debated, particularly in relation to the doses used in animal models. For example, recent studies used valproate doses of 7–33 times the maximum human dose equivalent [70]. Testicular atrophy is reported in rats given valproate at 13 times the human dose equivalents [71]. Other valproate rodent studies report reversible abnormalities in sperm count and motility [70]. Fertility was unchanged in male rats given up to 500 mg/kg/day (17 × maximum human dose), but sperm motility and weights of epididymis, seminal vesicles and prostate were decreased. At higher doses, of 1000 mg/kg/day—equivalent to human dose of 70,000 mg daily (> 33 times the maximum human dose) the rats were moribund or dead at day 5 of the experiment [70].

Sperm and semen quality, morphology and quantity fluctuate significantly in all males and is affected by mood, stress, season, age, ethnicity and lifestyle factors (including alcohol, cigarettes, cannabis, exercise and even choice of underwear) in males without and with epilepsy [72].

There are reports of abnormalities in semen analysis, lower sperm count and motility, and dysmorphic sperm in men with epilepsy before starting ASMs [73]. Similar findings are seen in men prescribed carbamazepine, oxcarbazepine, phenobarbital, phenytoin, valproate, and levetiracetam (Grade B &C) [34]. Lamotrigine has the least reported impact on spermatogenesis [73], with little data on the newer ASMs.

There is Grades B–D evidence of abnormalities in sperm count, morphology and motility in men taking valproate [34, 74–77]. Reduced testicular volume and sperm abnormalities were reported in 27 men treated with valproate compared to 41 controls [73] with similar findings in another study of 25 men [74]. A case study, without controls or pre-treatment values found a reversible reduction in sperm count in a man taking valproate [77]. Oxcarbazepine, levetiracetam and lamotrigine had no significant effect on sexual function and sex hormones in a study of 38 men. In fact, oxcarbazepine improved sperm motility and survival rate [78]. In 26 men taking levetiracetam, total sperm count, percentage of normal morphology and functional sperm count tested after treatment were significantly lower compared with pre-treatment values (*p* < 0.05), but there were no effects on hormone levels [79]. There is little data on the newest ASMs.

### Genetic and transgenic effects

Tests for gene mutations related to valproate in sperm cells are negative, therefore, transmission is unlikely. Some tests for severe chromosome damage were positive which would lead to sperm cell death and thus no transmission of mutations to offspring. Whether milder chromosomal damage might be transmitted to the offspring is unknown [69].

Epigenetic mechanisms for example via DNA methylation have been postulated as a mechanism for transgenerational passage of autism [79, 80]. A rodent study proposed it as a potential mechanism for teratogenic effects of valproate in second and third generation and increased levels of autism [80]. Study limitations are valproate doses equivalent to 12 × human dose (28000 mg), and small sample size (4 dams in each group). There was no testing of generation 0. The parents of the first generation of mice offspring were not controlled in comparison to their male offspring, only male offspring were studied. The findings also included a reduction in anxiety in first, second and third generation offspring, which was not explained or discussed in the conclusion. Researchers found a lowered threshold in the electroshock model (used as a surrogate for seizures), and they imputed that the male mice with mothers exposed to valproate had an increased risk of seizure. This is both biologically implausible and never described in other models or in humans.

Another study failed to replicate transgenerational effects in mice [79]. Valproate administration induced histone hyperacetylation in testes, but this effect was transient and did not translate to the next generation. Doses used of 200 mg/kg were equivalent to 14,000 mg daily (6 × the usual human maximum dose). Behavioural testing showed a change in light–dark behaviour suggestive of reduced anxiety (not autistic-like behaviour) in the first generation. The authors concluded that valproate treatment relaxes chromatin and increases susceptibility to epigenomic changes but does not produce DNA methylation changes that would persist for multiple generations.

## Population studies

There have been recent concerns about a possible increase in major congenital abnormalities and neurodevelopmental disorders (intellectual disability (ID); Communication disorders; Autism Spectrum Disorder (ASD); Attention–Deficit/Hyperactivity Disorder (ADHD); Neurodevelopmental motor disorders, including Tic disorders; and Specific learning disorders) [81] in offspring of males taking valproate. Population studies provide large datasets which can inform this question, but caution is needed to avoid conflating association with causality, assessing homogeneity of cohorts, and critical granularity is lacking [82].

In a nationwide, Swedish registry study of > 1 million births 4544 births were recorded to 2955 fathers with epilepsy, of whom 45.9% were prescribed ASMs during conception. There was no increased risk of MCM, autism, ADHD or intellectual disability compared with offspring of fathers with epilepsy not exposed to ASMs. Rates of autism were 2.9 per 1000 child-years and intellectual disability was 1.4 per 1000 child-years in the offspring of fathers with epilepsy on valproate monotherapy. These were slightly higher compared with the offspring of fathers with epilepsy who did not use ASMs, but the propensity score adjusted analyses showed no statistically significant increased risk of adverse outcomes [83].

A Norwegian prescription database of 340 000 pregnancies occurring between 2004 and 2010 investigated offspring outcomes from fathers and specific drugs dispensed during 3 months prior to conception. 26% of the fathers were dispensed one or more drugs and 1.3% were dispensed at least one drug “requiring special attention.” Adverse pregnancy outcomes were generally not increased [OR 0.99 (95% CI 0.94, 1.0)]. Valproate was not associated with increased risk [84]. The exception was fathers dispensed diazepam who had increased risks of perinatal mortality and growth retardation, with OR and 95% CIs of 2.2 (1.2, 3.9) and 1.4 (1.2, 1.6), respectively. Highlighting the pitfalls of equating correlation and causation [82] further analysis uncovered that diazepam use in males correlated with smoking and co-diazepam use in their female partners. Removing this confounder eliminated any correlation between male use of diazepam and increased risks in offspring [85].

A 2019 Danish population-based study in 733, 282 males found a 23% increase in congenital malformations in offspring of fathers using ASMs [86]. When extending the exposure window to 1 year before conception to the end of pregnancy, except for those using ASMs during the 3 months before conception (the susceptible period of exposure), the increased risks were also observed in children whose fathers were former users (i.e., those using ASMs only from 1 year to 3 months before conception) (OR 1.29, 95% CI 1.03–1.61) and later users (i.e., those using ASMs only during pregnancy after conception of their child) (OR 1.35, 95% CI 1.12–1.65). This study suggested that the increased risk of congenital anomalies in the offspring associated with paternal ASMs use before and after conception may be attributable to the underlying indications related to ASMs use; and unlikely to be ascribable to ASMs.

## Ethics of patient choice

The discrepancy between advice about the first-choice treatment offered to men and women arising from previous MHRA guidelines whereby men with generalised tonic–clonic seizures were offered valproate as "first-choice" and women were not, created legal and ethical challenges. Health care professionals were aware of their ethical obligations to treat a patient based on clinical need and within the Equality Act (or equivalent legislation outside the UK). There were major implications in exposing only women to a higher risk of seizures resulting from use of less efficacious medication.

The new January 2024 MHRA regulation removes this difference for all people under the age of 55 years [26], now exposing individuals with epilepsy to a higher risk of seizures. While seen as a solution to address the gender gap, this brings considerable new challenges to prescribers while still not addressing some existing concerns.

Both the new and previous MHRA regulation and it’s recording systems—the RMMs—conflict with a range of ethical–legal issues:

- The *Montgomery* and *McCulloch* rulings.
- GMC Guidance on informed consent.
- Article 2 of the European Convention on Human Rights (ECHR)
- The  common law principle of English Law which holds  that patient autonomy takes precedence over concern for the health of a future child

### The *Montgomery* and *McCulloch* ruling

The RMM guidelines do not address the legal challenge encapsulated in judgment in *Montgomery v Lanarkshire Health Board* [2015] [87]. The medical professional must take reasonable care to ensure that the patient is aware of any material risks involved in any recommended treatment—but also any suitable alternative or variant treatments [2, 6, 87].

The question of “who decides” which alternative treatments are suitable was recently considered in *Bilal & Anor v St George’s University Hospital Trust* [2023] [88] and *McCulloch and others v Forth Valley Health Board* [2023] [89]. Both the Court of Appeal and Supreme Court held, respectively, that this decision is a matter of “*professional skill and judgment to which the professional practice (Hunter v Hanley/Bolam) test should be applied.*” Further, the Court in *Bilal* emphasised the importance of the specific medical speciality when determining alternative treatments. These judgements mean that the option of treatment with valproate must be discussed with everyone who presents with responsive seizure types, particularly if it is likely to be the most efficacious treatment for them [31].

The law is clear on the role of clinicians on such matters. It is not satisfactory to simply present the patient with technical information “*which she cannot reasonably be expected to grasp, let alone by routinely demanding her signature on a consent form*” (*Montgomery* [2015] UKSC 11 at 90) [87].

Yet regulatory body information leaflets for patients [20–26] do not outline the risks of stopping valproate estimated as a 30–40% chance of recurrence or worsening of seizures [90, 91]. Neither do they explain the risks of “user independent” contraception, nor the risks and benefits of the alternative treatments. Thus, the MHRA valproate regulations do not meet requirements for patient decision-making in line with the *Montgomery* ruling regarding disclosure of ‘material risks’[6, 87].

### GMC guidance on informed consent

MHRA regulations also appear to conflict with the GMC guidance on treating individuals who can make their own decisions, in partnership with their doctors. The GMC and the law respect the fact that patients in their decision-making process can retain the right to make “unwise decisions” or decisions their doctors may not agree with [93]. Following the "GMC Decision Making and Consent Professional Standards"(2020: principle 4, reference): “*doctors must try to find out what matters to patients so they can share relevant information about the benefits and harms of proposed options and reasonable alternatives, including the option to take no action*.” Guidance includes that doctors should explore:

- *“what matters to patients about their health—their wishes and fears, what activities are important to their quality of life, both personally and professionally—so you can support them to assess the likely impact of the potential outcomes for each option.*
- *“with patients what risks they would and wouldn’t be prepared to take to achieve a desired outcome, and how the likelihood of a particular outcome might influence their choice*

The current MHRA guidance puts clinicians in a bind as based on medical expertise, valproate is a ‘reasonable alternative’, as the most effective medication for some epilepsies [6, 9–13]. In prescribing this, potentially off-licence if MHRA requirements are not met irrespective of the impact on people with epilepsy themselves, prescribers may fall foul of the MHRA. In not prescribing valproate for an informed patient opting for it, they fail the patient, as well as their legal and GMC obligations.

### Article 2 of the European Convention on Human Rights (ECHR) [93]

This specifies “a positive duty to prevent foreseeable loss of life.” Alternatives to valproate are likely to be less effective for a significant number and, therefore, carry greater risk of sudden death. At the minimum, this must be discussed with patients and their carers. Patients who elect to avoid valproate require closer monitoring, opportunities for urgent review and the right to change to valproate treatment. For some, this is too late, and SUDEP has occurred in an unknown and unmonitored number of people avoiding valproate.

### The common law principle of English Law [94]

This holds that an individual with capacity can accept or refuse treatment—even if it is against medical advice or not in the best interest of a possible future child. The law is clear that a competent woman who has the capacity to decide may, for religious reasons, other reasons, or for no stated reasons at all, choose not to have medical intervention, even though, the consequence may be the death or serious disability of the child she bears, or her own death. The foetus up to the moment of birth does not have any separate interests capable of being taken into account when a court has to consider an application for a declaration in respect of a caesarean section operation. The court does not have the jurisdiction to declare that such medical intervention is lawful to protect the interests of the unborn child even at the point of birth. [95](Re MB [1997] EWCA Civ 3093 (following statement made in support of an earlier case—Re T [1992] 4 All ER).

The MHRA’s stated aim is preventing any pregnancies in parents taking valproate, focusing on foetal risk [6, 11, 13, 96]. This treats a valproate exposed pregnancy as a “never event” and protects against certain future legal claims (Box 2) [96]. The assumption that valproate usage is “preventable” enforces a certain definition, which is not supported by the science. This raises questions around the moral and legal status of the “hypothetical foetus” especially if medication is restricted among people who are not actively planning pregnancies. Current MHRA regulation means that the prevention of potential harm to a hypothetical foetus takes priority over ensuring the availability of effective life changing medication and implies that all individuals for valproate purposes should always be treated as “pre-parents” [6, 11, 96–99].

The recent Confidential Inquiry into Maternal Deaths [97] has identified an increase in the deaths of women with epilepsy during pregnancy, and without specifically attributing this to changes in use of valproate in this population the authors do say: “*It is of concern that current discourse around valproate use remains focussed around fetal risk without the essential focus on ensuring that women receive alternative effective anti-epileptic medication*.”

## Discrimination in reproductive rights

In many countries, people may make reproductive decisions with a wide range of options. For example, a person with a serious autosomal dominant genetic condition may choose to have a child without any intervention, or have a termination if they get pregnant, or have pre-natal testing if that is available [6]. They are not required to use contraception, nor told what type of contraception they must use, in order to obtain optimal medical care. They may decline to discuss their sexuality with their healthcare professionals if they wish. People choosing valproate as their treatment are denied these rights [6, 96, 98, 99]. In addition, they now, as per the new MHRA guidelines, may face the stress of potentially running out of their life-saving medication if the necessary reviews and dual signatories are not completed in time [15, 99].

Box 2: Consensus and assumptions on valproate use *(adapted from Angus-Leppan & Liu, 2018)* [6] **Consensus**

 Valproate has significant teratogenicity in women.

 The teratogenicity of some alternatives to valproate are unknown.

 All women of childbearing potential taking valproate need expert advice, information about its risks, and regular review.

 People taking valproate should not stop taking it suddenly but seek specialist medical advice.

**Assumptions by those supporting prohibition of valproate.**

 Valproate is a major teratogen and this consideration over-rides all others.

 Risks of seizure breakthrough and SUDEP are less important than teratogenicity.

 Polytherapy with other drugs is less harmful than valproate.

 There are always efficacious alternatives to valproate and these are lower risk to the fetus.

 Children should not take valproate even before parenthood is a possibility because of the risk that they will continue it into child producing years.

 Women who wished to become pregnant would not take valproate if they knew the risks.

 Women taking valproate should not be permitted to become pregnant.

 Parents would rather have no child than a disabled child.

 Men wishing to father children expect zero risk of teratogenicity.

 Slow reactivity to teratogenicity of valproate in women means that any risk of teratogenicity in men requires immediate action.

**Assumptions made by those supporting informed choice.**

 Individuals have the right to choose valproate treatment after informed discussion.

 Some patients will place personal safety considerations over concerns about teratogenicity, and this should be respected.

 Some patients would prefer to continue valproate during parenthood.

 Alternative treatments to valproate are not risk free.

 Assessment of the risk–benefit analysis for valproate is an individual judgment.

 In some situations low dose valproate may be lower risk than high dose polytherapy.

 Not everyone wants to have children, and those who want children do not want to be having children all the time.

## Circumstances when a patient or representatives elects for valproate and not the Pregnancy Prevention Programme (PPP)

There are situations in which a child or adult or their authorised representative may decide that she will continue valproate even though she might be, or may become, pregnant, and when the PPP is not appropriate (Box 3).

### Patients may elect for valproate as their preferred treatment option, but decline PPP

People may make this decision de novo, if evidence suggests that valproate is likely to be the most effective medication. They may wish to avoid trials of less effective medication, to reduce the risk of injury, disruption to their life, employment and driving, and SUDEP (1/500 on average, 1/100 for refractory epilepsy) [100]. Patients may undergo one or more unsuccessful trials of other medications in line with MHRA guidelines to avoid valproate, and then opt for valproate treatment. Symptom free patients may wish to remain on valproate to avoid the risks to their personal safety of seizure recurrence in changing to another medication, which survey evidence (4774 patient encounters by 215 clinicians) [90] and a population study [91] suggest is in the order of 30–40%. There are little data on the long-term effects of exposure of babies, infants and children to uncontrolled seizures during their period of maximum neural development, but Grade C evidence suggests that it may damage cognitive development [6]. Some parents or guardians may elect for their child to be on valproate without contraception to optimise seizure control, safety and learning.

After informed discussion, people may decline the contraception recommended by the MHRA for personal, religious or health reasons. For some people sexual relationships outside marriage are forbidden for religious or cultural reasons and using any form of contraception before marriage is thus unacceptable. Other people who do not have heterosexual sex, i.e., who are abstinent, celibate, asexual (ace), or in same-sex relationships may find the PPP unnecessary and unacceptable, and probing about their sexuality intrusive and undignified. Women who are not able to become pregnant for health‐related or physical reasons will not wish to have “user independent” contraception, and repeated discussions as currently stipulated by the PPP when the situation is unchanged, may find the whole experience unpleasant and superfluous. Some women will prefer other contraception.

### Intellectual disability (ID)

Systematic reviews estimate that 22% of people with ID have epilepsy, and prevalence rises with the increasing severity of ID [101]. Two-thirds of the population with ID and epilepsy are treatment resistant [102]. Only 5% of research in epilepsy focuses on ID and many clinical trials in epilepsy exclude people with ID [103, 104]. People with ID and epilepsy have significantly higher multi-morbidity, polypharmacy and higher rates of premature mortality than their peers with epilepsy or intellectual disability [105]. Further, SUDEP and epilepsy related harm is considered to be of higher risk in this population [105–107]. A third of People with ID and epilepsy suffer psychiatric comorbidities [105–108].

A Cochrane review recommended using the same treatment paradigms for people with epilepsy and ID as the general population [109], however this is an area which has a poor evidence base especially given the other issues. An expert consensus study suggested that valproate should be considered as first line drug in men with ID [110]. Valproate is often a good choice given its additional mood-stabilising effects, and in those with autism, thus reducing polypharmacy. It has been shown to be the most prescribed ASM in international comparison studies [110–112]. Valproate remains a first-line drug for generalised seizures and people with ID and a number of genetic syndromes are specifically responsive [112, 113]. As valproate is a mainstay of treatment in this population, excluding it as a treatment option puts many vulnerable individuals at significant risk of harm.

People with moderate to profound intellectual disability (ID) will usually lack the mental capacity to consent to sexual relationships and pregnancy in this population would raise serious safeguarding concerns, implying sexual abuse [9, 10, 12]. “User independent” contraceptives may put women in this population at unnecessary risk. There may be other clinical indications for contraceptive use such as dysmenorrhea; therefore, each case needs to be considered on an individual basis following the best-interests process under the 2005 Mental Capacity Act (MCA) or local equivalent [9–12]. Repeated discussions on this topic mandated by a yearly consent form can be an unnecessary and distressing process for the individual and their families [6, 9, 12].^9B^

The default position is always to assume people have mental capacity [9, 12, 98]. Those with mild ID should be managed as such unless there is clear evidence otherwise. They may need extra time and additional support from carers in their decision making, and possibly augmented communication. In this there is a significant concern that older people with ID and epilepsy would lack suitable consistent representation. It has been evidenced that their epilepsy care is poorer than their younger peers while needs are greater [113].

### Emergency situations

In emergencies such as status epilepticus or serial seizures, discussion with the patient is usually not possible and treatment is given on a best interest basis under mental capacity legislation [9, 10]. Emergency care should never be delayed because of potential teratogenicity or pregnancy. The MMBRACE report highlights fatal outcomes caused by withholding life-saving treatments because of fears of harming the foetus [98]. Emergencies in pregnancy require proactive and same care as non-pregnant people. Informed discussions about the associated benefits and risks of continuation of valproate would need to be undertaken on recovery with the person, or their advocates if capacity is impaired [6, 9, 10, 101, 112].

Recommendations (see Table 2).

**Table 2:** Recommendations

Suspension of January 2024 MHRA guidelines pending further review
Full disclosure of the CHM & MHRA proceedings regarding valproate
Revised MHRA information packs, which include risks and benefits of valproate, alternatives and user independent contraception
Acknowledgement of the full range of patient lifestyle choices and sexual preferences in guidelines
Funding for additional clinical time for informed patient decision-making
Government funded prospective registries of all maternal & foetal outcomes of all pregnancies in epilepsy and bipolar disease
Government funded registry of patients switching from valproate to other medications
Full disclosure of the CHM & MHRA funding & Disclosures (individual & organisational)
Full & free availability of all reproductive options (including egg harvesting, termination, surrogacy, adoption, sperm banking) for people wishing to commence valproate

### Full disclosure by the MHRA

The MHRA should disclose all information and the Minutes of discussions about valproate and all medications under their scrutiny. It is a concerning precedent that they have made a major decision influenced by unpublished materials especially as they may have not been peer-reviewed. There is acknowledgement that the study was flawed but needs to be factored to the decision currently held. Disclosures and potential conflict of interest (both professional and personal) of individual members of the MHRA committees and the funding sources for the MHRA should be made freely available to the public.

### Adequate resources for informed patient choice and outcome monitoring

In the USA, there were 3 million valproate prescriptions in 2021 [115]. In 2021, the MHRA estimated there were ~ 20,000 females and ~ 50,000 males taking valproate in the UK [7]. Many of the adult men on valproate are not under specialist care, as they are seizure free. Primary care professionals are now under pressure to refer these patients for review, to already overstretched secondary care facilities. The MHRA has not provided resources for the task of identifying and reviewing these patients. This leaves patients vulnerable to running out of medication and to breakthrough seizures, not to mention the unquantified significant risk emerging on mental health issues. The projected 30–40% of seizure recurrences in those stopping valproate [90, 91] leaves these patients needing urgent medication review, at risk of SUDEP (1/500) [100], and unable to drive with implications for quality of life, employment and other responsibilities.

Adherence to medication regimens by people with epilepsy is fragile—although missing tablets is associated with a risk of morbidity and mortality/SUDEP, up to 79% of people may not take their medication as recommended [116]. Numerous factors are relevant, but among them are a patient's beliefs and understanding about the risks and benefits of the drug they are prescribed and logistical barriers to patients receiving regular scripts [99, 117]. To optimise adherence, a doctor must ensure that the messages about the medication and its use are communicated clearly. This cannot be done effectively without more resources, particularly clinician time.

Studies of outcomes of the decision-making regarding valproate are needed—these should be government funded and relate to all patients considering valproate and alternatives, and those switching to other medications.

### Reversion to 2016 international guidelines

The 2016 Joint Task Force guidelines of the International League Against Epilepsy, Commission on European Affairs, and European Academy of Neurology (adopted in China and elsewhere) focus of informed decision-making by the patient, balancing the risks and benefits for the individual patient.

## Conclusions

There is consensus on the teratogenic harms of valproate for pregnant women and the risk of neurodevelopmental disorders in offspring. The patients and families harmed by valproate, and those who were not warned about these, are outraged. They deserve full compensation and financial and social support, as do all vulnerable and disabled people in societies promoting equality and social justice.

The impact of valproate on male fertility and possible epigenetic effects are unknown, and the MHRA has refused to release the unpublished information on which they have based their restrictions in men. The UK is the only country with such restrictions. They do not align with the *Montgomery* and *McCulloch* judgements, GMC regulations or common law principles.

Recent regulations imposed by the MHRA effectively limit or prevent people from choosing valproate, even in circumstances when it is the only medication to prevent life-threatening seizures. 30–40% of people with epilepsy coming off valproate are predicted to experience seizures. Deaths have occurred because of the avoidance of valproate, and more will, unless the regulations are urgently amended. This article focused on adults with epilepsy, but there are also people with bipolar disorder or other mental health problems for whom valproate is the best medication to keep them well. They are often too unwell to advocate for themselves. Uncontrolled seizures, cognitive and neurodevelopmental consequences for young people and children denied valproate from birth have potential long-term and irreversible consequences. It is ironic that restrictions to protect future unborn offspring will harm the neurocognitive development and well-being of some living children.

Necessary elements to ensure patient safety include a return to the consensus valproate guidelines of 2016, resources for monitoring of patient outcomes, public scrutiny of the withheld reports and minutes of MHRA proceedings on valproate, and restoration of informed decision-making for people considering valproate as a treatment.

### Supplementary Information

Below is the link to the electronic supplementary material.

Supplementary file1 (DOCX 16 kb)
