# Supplementary material for: New valproate regulations, informed choice and seizure risk
Source: J Neurol. 2024 Jun 19;271(8):5671–86. doi: 10.1007/s00415-024-12436-8 (PMC11319429; doi:10.1007/s00415-024-12436-8)
Supplement: Supplementary file 1 — Supplementary file1 (DOCX 16 kb) [file 415_2024_12436_MOESM1_ESM.docx]

Supplementary information

BOX 3: case vignettes (anonymised, with patient consent)

*17-year-old woman with JME with tonic clonic seizures weekly. Previous unsuccessful trials of lamotrigine, levetiracetam, zonisamide, clobazam. Has lost 6 months of schooling in the last year, previous dislocated shoulder and injury to two front teeth during seizures. Does not want to be on an implant or Intrauterine contraception and she is not sexually active.*

*47-year-old man who does not want any children and does not want to discuss his sexual orientation. He has been on valproate and seizure free for 20 years, apart from tonic clonic seizure one year ago on attempting to reduce valproate. Does not want to discuss medication effects on sperm nor sign any PPP forms about his treatment.*

*23-year-old woman with profound learning disability and a genetic syndrome on valproate and lamotrigine. Behavioural problems while taking levetiracetam. She has been free of major seizures for 7 years. Her parents are unhappy to have to repeat the discussions about contraception each year, they say she could only get pregnant if she was raped, and that it is upsetting to have to waste time repeating the conversation each year as they have other important things to talk about with their epilepsy nurse and doctor, as they only get to see them every year now.*

*39-year-old woman with JME, seizure free for 20 years on valproate. She has a daughter aged 7 years. She would like another child and has been unable to conceive and would like to have IVF. She does not want to stop her valproate.*

*25-year-old woman, GGE with one tonic–clonic seizure weekly, depressed and unable to work. Multiple injuries to face, teeth and limbs during seizures. She has tried lamotrigine, perampanel, levetiracetam, clobazam, zonisamide and lacosamide without success. She would like to try valproate but would like to do egg harvesting prior. This is refused on the NHS, and she cannot afford this privately. She asks why it is not allowed on the NHS, when it is allowed for someone before chemotherapy. She is keen to have a child in the future.*
